# Cultivation reveals physiological diversity among defensive ‘*Streptomyces philanthi’* symbionts of beewolf digger wasps (Hymenoptera, Crabronidae)

**DOI:** 10.1186/s12866-014-0202-x

**Published:** 2014-07-29

**Authors:** Taras Y Nechitaylo, Martin Westermann, Martin Kaltenpoth

**Affiliations:** 1 Max Planck Institute for Chemical Ecology, Insect Symbiosis Research Group, Jena, Germany; 2 University Hospital, Centre for Electron Microscopy, Jena, Germany

**Keywords:** Streptomyces, Physiology, Symbiosis, Mutualism, Co-evolution, Beewolf, Digger wasp

## Abstract

**Background:**

‘*Candidatus* Streptomyces philanthi’ is a monophyletic clade of formerly uncultured bacterial symbionts in solitary digger wasps of the genera *Philanthus*, *Philanthinus* and *Trachypus* (Hymenoptera, Crabronidae). These bacteria grow in female-specific antennal reservoirs and – after transmission to the cocoon – produce antibiotics protecting the host larvae from fungal infection. However, the symbionts’ refractoriness to cultivation has thus far hampered detailed *in vitro* studies on their physiology and on the evolutionary changes in metabolic versatility in response to the host environment.

**Results:**

Here we isolated in axenic culture 22 ‘*Streptomyces philanthi*’ biovars from different host species. Sequencing of *gyrB* revealed no heterogeneity among isolates within host individuals, suggesting low levels of (micro)diversity or even clonality of the symbionts in individual beewolf antennae. Surprisingly, however, isolates from different host species differed strongly in their physiology. All biovars from the Eurasian/African *Philanthus* and the South American *Trachypus* host species had high nutritional demands and were susceptible to most antibiotics tested, suggesting a tight association with the hosts. By contrast, biovars isolated from the genus *Philanthinus* and the monophyletic North American *Philanthus* clade were metabolically versatile and showed broad antibiotic resistance. Concordantly, recent horizontal symbiont transfer events – reflected in different symbiont strains infecting the same host species – have been described only among North American *Philanthus* species*,* altogether indicative of facultative symbionts potentially capable of a free-living lifestyle. Phylogenetic analyses reveal a strong correlation between symbiont metabolic versatility and host phylogeny, suggesting that the host environment differentially affects the symbionts’ evolutionary fate. Although opportunistic bacteria were occasionally isolated from the antennae of different host species, only filamentous Actinobacteria (genera *Streptomyces*, *Amycolatopsis* and *Nocardia*) could replace ‘*S. philanthi*’ in the antennal gland reservoirs.

**Conclusion:**

Our results indicate that closely related bacteria from a monophyletic clade of symbionts can experience very different evolutionary trajectories in response to the symbiotic lifestyle, which is reflected in different degrees of metabolic versatility and host-dependency. We propose that the host-provided environment could be an important factor in shaping the degenerative metabolic evolution in the symbionts and deciding whether they evolve into obligate symbionts or remain facultative and capable of a host-independent lifestyle.

## Background

Mutualistic associations between invertebrate hosts and bacteria are widespread in nature [1] and have important implications for host ecology and evolution [2]. While the taxonomic and functional diversity of bacterial symbionts has been – and continues to be – studied extensively, particularly in insects, the fastidious nature of most symbiotic bacteria and their refractoriness to axenic cultivation [3] has in most cases hampered detailed investigations of the symbionts’ physiology and the molecular underpinnings of symbiosis establishment through targeted genetic manipulation (but see [4]–[7]).

Most insect-bacteria symbioses have a nutritional basis, with Proteobacteria, Firmicutes, and Bacteroidetes as especially common and widespread symbionts providing limiting nutrients to their hosts [8]. However, more and more defensive alliances for the host’s protection against parasitoids, predators, and/or pathogens are being discovered [9],[10], and filamentous Actinobacteria are especially prevalent as protective symbionts, due to their ability to produce a range of bioactive secondary metabolites [11],[12]. Antibiotic-producing Actinobacteria have been implicated in pathogen defense of fungus gardens or galleries in leaf-cutter ants [13]–[15] and bark beetles [16], respectively, as well as in the protection of the developing offspring in solitary beewolf wasps [17]. Additionally, Actinobacteria have been isolated from mud-dauber wasps [18], termites [19], the nests of *Allomerus* ants [20], and several other insect taxa, but their possible involvement in the protection of the hosts remains to be investigated.

Of all protective actionbacterial symbionts, *‘Candidatus* Streptomyces philanthi’ constitutes so far the only known specific *Streptomyces* symbiont tightly associated with an insect. These bacteria populate female-specific antennal gland reservoirs of solitary digger wasps of the genera *Philanthus*, *Philanthinus* and *Trachypus* (Hymenoptera, Crabronidae, tribe Philanthini) [21],[22], where the host provides its symbionts with nutrients [23],[24]. Similar to the symbiotic Actinobacteria of leaf-cutting ants [13], *‘Ca.* Streptomyces philanthi’ plays a defensive role in symbiosis: after secretion of the bacteria from the females’ antennae into the subterranean brood chambers, the larvae apply the symbionts onto the cocoon surface, where within a short (1–2 weeks) period the bacteria produce a ‘cocktail’ of two different groups of antibiotics, streptochlorin and several piericidin derivatives, thereby protecting the larva from fungal infection during the vulnerable phase of the host’s hibernation [17],[25]–[27].

Recent phylogenetic analyses revealed that the symbiosis between beewolf digger wasps and protective *Streptomyces* bacteria already evolved in the late Cretaceous (at least 68 million years ago) [28]. Over the long evolutionary timescales, the association was stabilized by a combination of partner fidelity through vertical transmission and partner choice by host control over symbiont transmission [28]. The high degree of specificity in this intimate relationship resulted in a consistent association with a single clade of *Streptomyces* across Philanthini wasps.

Long-term intimate symbiosis often leads to host-dependency of the symbionts due to genome erosion [29],[30]; concordantly, most microbial symbionts cannot be isolated in axenic culture by traditional techniques [3]. Unlike the above-mentioned Actinobacteria of leaf-cutting ants, this is also true for ‘*Ca*. Streptomyces philanthi’, which seems to have lost certain metabolic capabilities during the long time of association with its host [21]. Its refractoriness to cultivation so far prevented insight into their physiology as well as into host-symbiont interactions in the antennal gland reservoirs, specifically nutritional benefits provided by the host.

Here we report on the isolation and axenic cultivation of symbiotic *Streptomyces* from 22 beewolf host species comprising all three Philanthini genera collected over a broad geographic range (Eurasia, Africa, North and South America). *In vitro* cultivation allowed us to address three important questions in the context of the symbiotic association with Philanthini: (i) How does the physiology of beewolf symbionts differ from free-living relatives? (ii) Do the symbionts of different host species share the same physiological characteristics including nutritional requirements, indicating host-symbiont co-adaptation in the early stages of the symbiosis? (iii) Are host individuals colonized by a single symbiont strain in the antennae? The results are discussed in light of the evolutionary history of this defensive symbiotic association.

## Results

### Isolation of ‘*Streptomyces philanthi* biovar *triangulum*’

**Figure 1:**
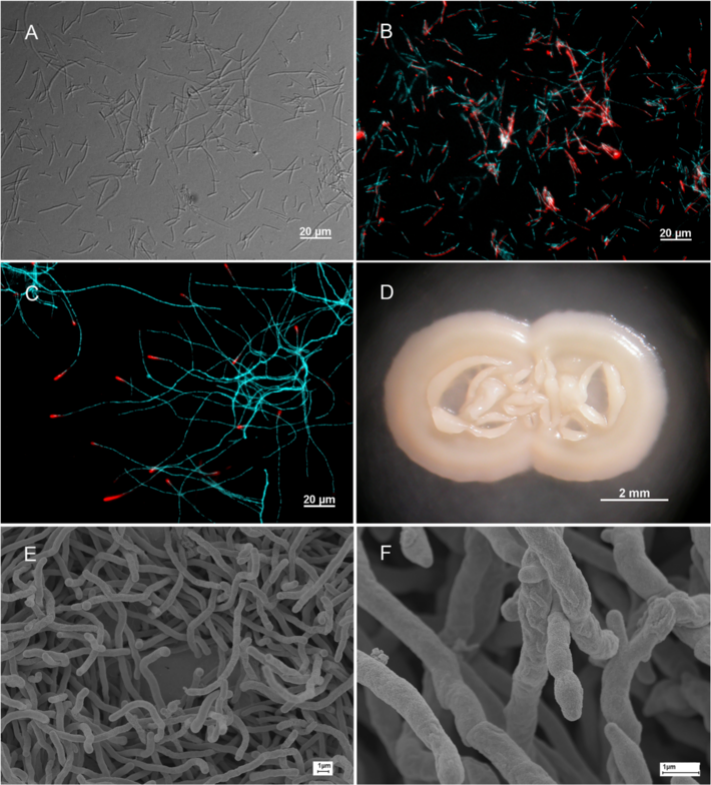
**Morphology of ‘*S. philanthi*biovar*triangulum*’. (A)** Differential interference contrast (DIC) micrograph of ‘*S. philanthi* biovar *triangulum*’ in an antennal sample. **(B)** FISH micrograph of the same area as shown in A, with the ‘*S. philanthi*’-specific probe Cy3-SPT177 (red), and DAPI for unspecifically staining bacterial DNA (blue). **(C)** FISH micrograph of a pure culture of ‘*S. philanthi*’ with Cy3-SPT177 (red) and DAPI (blue). **(D)** Colony of ‘*S. philanthi*’ grown on the solid Grace’s medium. **(E, F)** Scanning electron micrographs of aerial mycelium from matured ‘*S. philanthi*’ colonies grown on the solid Grace’s medium.

Due to the availability of a laboratory colony of *Philanthus triangulum* and an ongoing genome sequencing project of its symbionts, the isolation of ‘*Ca*. Streptomyces philanthi biovar triangulum’ was of our specific interest. In preliminary experiments, this bacterium did not grow on ‘standard’ (and relatively simple) nutrient media (R2A and Actinobacteria isolation agar) (see also [21]). Therefore, we used Grace’s insect medium (Additional file 1: Table S1 and Additional file 2: Table S2), which might imitate, to some extent, antennal gland exudates or insect hemolymph – the most likely source of nutrition in the natural habitat of the bacteria in the beewolf’s antennal gland reservoirs. Because the composition of beewolf hemolymph and gland secretions were unknown, other supplements (fetal bovine serum (FBS) and mammalian cell lines media) were added to increase the availability of compounds in the nutrient media. In antennal samples prepared for inoculation, ‘*Ca*. Streptomyces philanthi’ looked like individual or relatively short-chained unbranched cells; long mycelium, typical for free-living members this bacterial genus, was very rare (Figure 1A). FISH analysis demonstrated that the majority of these bacterial cells were physiologically active (Figure 1B).

In complex liquid media, the bacteria formed typical streptomycetal mycelium with terminal physiologically active cells (Figure 1C) and grew as polymorphic (often irregular but also round, sometimes even ribbon-like) colonies. Despite this polymorphism, the sequence analysis confirmed the purity of the cultures – analyzed amplicons of 16S rRNA, *gyrA* and *gyrB* gene fragments were identical to the respective sequences of ‘*Ca*. Streptomyces philanthi biovar triangulum’. According to the classification rules for cultured and uncultured prokaryotes [31], the isolated but not yet characterized bacterium was designated – and is hereafter referred to – as ‘*Streptomyces philanthi* biovar *triangulum*’ strain tri23Af2. On solid media, the strain tri23Af2 formed beige opaque colonies of slightly shiny surface varying from smooth to rimmed and rugose (Figure 1D); typical streptomycetal colonies with fuzzy surface formed by aerial sporulating hyphae were not observed even after long incubation (1 month at 28°C plus 3 weeks at 10-14°C) (Figure 1D). Likewise, scanning electron microscopy of mature colonies grown on solid Grace’s medium did not reveal spores (Figure 1E-F). Apparently, these symbionts have either lost the ability to form spores, or sporulate only under *in vivo* conditions and would need specific stimuli to do so *in vitro*.

**Figure 2:**
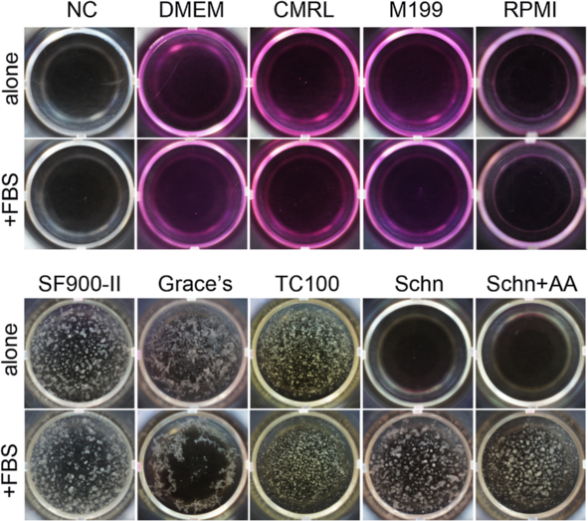
**Growth of ‘*S. philanthi*biovar*triangulum*’ strain tri23Af2 in different media.** Media were either supplemented with (+FBS), or not (alone). (NC): negative control (1× PBS); (Schn): original Schneider’s *Drosophila* medium alone and with missing amino acids added (Schn + AA). Bacteria were grown at 28°C for 7 days.

Strain tri23Af2 showed the best growth in the medium SF900-II (Gibco). However, other insect media (Grace’s and TC-100 alone and with 10 % FBS) or Grace’s-based medium M522 were also suitable for cultivation (Figure 2); additionally, it grew in the media M252 and M225 (Additional file 1: Table S1), but with lower growth rates than in Grace’s medium (data not shown). Surprisingly, the strain tri23Af2 did not grow in the original Schneider’s *Drosophila* medium alone, even though the composition and pH of this medium was very similar to other insect cell line media (Additional file 2: Table S2); moreover, further experiments demonstrated that Schneider’s *Drosophila* medium supplemented with missing amino acids (L-alanine, L-asparagine and L-phenylalanine; concentration as in Grace’s medium) was not suitable for symbiont cultivation either (Figure 2). However, FBS added to the Schneider’s medium could enable the growth of strain tri23Af2 (Figure 2). Interestingly, media designed for mammalian cell lines (DMEM, CMRL, RPMI and M199) alone or with FBS were also not suitable for the biovar ‘*triangulum*’ (Figure 2), even though these media are nutritionally rich and supported the growth of other bacteria including free-living *Streptomyces* (data not shown). Unfortunately, due to the complexity of the required nutrient media, we could not define which host-provided compounds were essential for growth of the biovar ‘*triangulum*’.

### Isolation and phylogenetic analysis of ‘*S. philanthi*’ biovars from other host species

**Figure 3:**
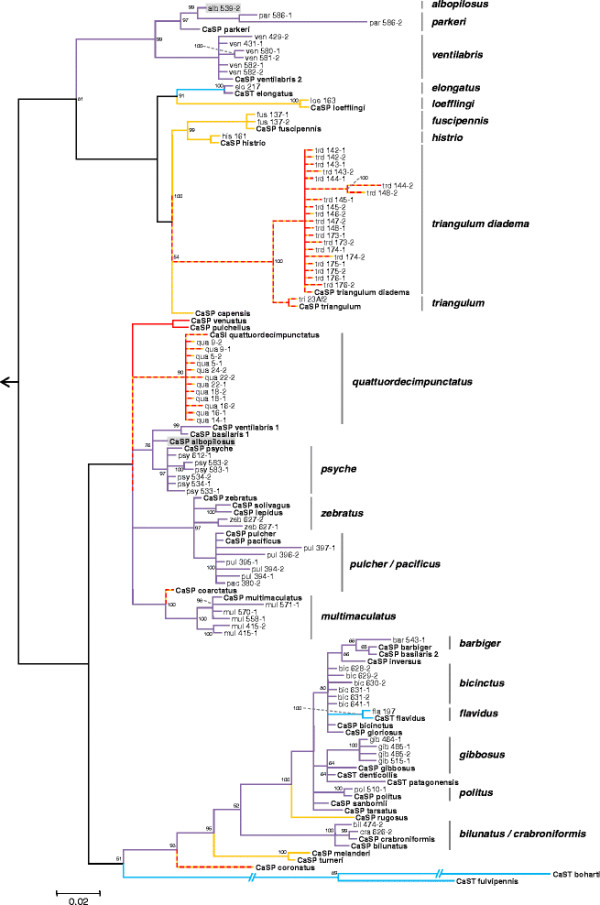
**Phylogenetic analysis of ‘*S. philanthi*’ isolates in respect to the sequences obtained from field-collected antennal samples.** Antennal isolates are indicated by their strain designation as explained in the Methods section (first three letters indicate host species), and the respective host species is additionally given behind each clade. Sequences directly obtained from beewolf antennae are indicated by “CaSP” and were obtained from a previous study [28]. The tree was reconstructed using nearly complete 16S rRNA genes and 660 bp-long *gyrB* gene fragments; values at the nodes indicate Bayesian posterior probabilities. Geographic distribution of beewolf taxa and the origin of isolated symbionts are indicated by branches of different colours on phylogenetic tree: Africa (yellow), Europe (red), mixed African/ Eurasian distribution (dashed yellow/red line), North and South America (purple and blue, respectively). Bacteria used as outgroups to root the tree are indicated in Additional file 4: Table S4. The discrepant phylogenetic placements of *Philanthus albopilosus* symbiont sequences from clones and isolates, respectively, are highlighted by grey boxes.

**Figure 4:**
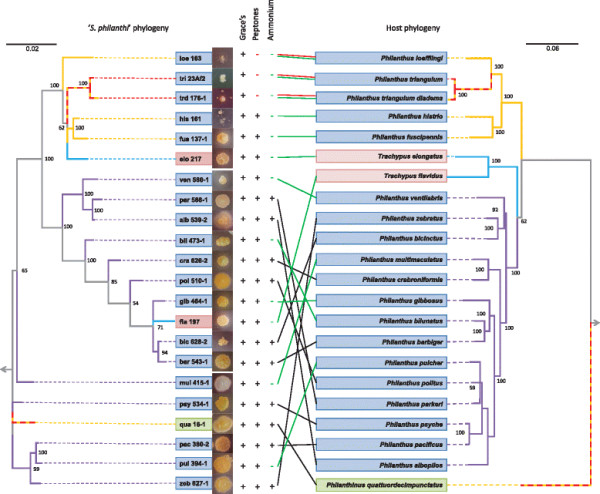
**Phylogeny of ‘*S. philanthus*’ biovars in respect to their morphology, nutritional requirements and host phylogeny.** The phylogeny of bacterial symbionts was reconstructed using nearly complete 16S rRNA genes, as well as *gyrA* and *gyrB* gene fragments (566 and 660 bp in length, respectively). The host phylogeny was obtained from [28]. Colored boxes around host and symbiont names denote host genera (green, *Philanthinus*; blue, *Philanthus*; red, *Trachypus*). Values at the nodes of the phylogenetic trees indicate Bayesian posterior probabilities. Geographic distribution of beewolf taxa and the origin of isolated symbionts are indicated by branches of different colours on phylogenetic trees: Africa (yellow), mixed African/Eurasian distribution (dashed yellow/red line), North America (purple) and South America (blue). Metabolic capabilities are indicated next to representative pictures of symbiont growth *in vitro*: Plus or minus indicate the ability (+) or inability (−) to grow on the corresponding media. In order to visualize the phylogenetic placement of symbionts and highlight their metabolic capabilities, symbiont strains were connected to their respective hosts with colored lines: Red lines correspond to strains unable to grow on medium with peptones; green lines correspond to strains unable to grow on ammonium as the only source of nitrogen.

For the isolation of additional ‘*S. philanthi*’ biovars, Grace’s insect medium with 10% FBS and cycloheximide (100 μg/ml) was applied. Overall, 22 biovars of the clade ‘*Streptomyces philanthi*’ were obtained from 23 host species. In some cases, antennal specimens did not yield culturable bacterial symbionts, or opportunistic bacteria grew instead (e.g. in the only specimen of *P. capensis*) (Additional file 3: Table S3). As had been observed previously [28], phylogenetic analyses based on 16S rRNA, *gyrA*, and *gyrB* sequences from symbiotic and free-living Actinobacteria demonstrated that the symbiont clade is monophyletic (Figure 3, Additional file 4: Table S4). Generally, the isolates clustered together with symbiont sequences obtained directly from the antennae of field-collected specimens of the corresponding host species. However, the strain alb539-2 of biovar ‘*albopilosus*’ affiliated to the biovars ‘*parkeri*’ and ‘*ventilabris*’ instead of the representative sequence of its own biovar (Figure 3). Analyses based on 202 AFLP markers were completely congruent with the sequence-based trees, supporting the robustness of the phylogenetic analyses and the displacement of strain alb539-2 (Figure 3, Additional file 5: Figure S1). A comparison of the symbiont phylogeny with a previously published phylogeny of the hosts based on one mitochondrial and five nuclear genes supported earlier findings of frequent horizontal transfer of symbionts among host species over evolutionary timescales (Figure 4) [28].

### Characterization of ‘*S. philanthi*’ biovars

In all antennal samples used for isolation, the symbiotic *Streptomyces* showed a characteristic “antennal” phenotype: bacteria looked like individual or relatively short-chained cells, unbranched or with very short side branches, while no well-developed long mycelium was observed (exemplified by biovar ‘triangulum’ in Figure 1A). In culture, the vast majority of biovars developed typical mycelium. However, two biovars were clearly distinguishable from all other symbiotic *Streptomyces* due to their “antennal” phenotype also in culture: in liquid medium, the actively growing biovar ‘*elongatus*’ formed micro-colonies, but in late stage of logarithmic growth or in stationary phase they tended to fall apart into short, often poorly branched fragments. A similar pattern was also observed for the biovar ‘*loefflingi*’, which could express one or the other phenotype over several transfers and change it by the next passage, although conditions triggering such phenotypic changes remained unknown (Additional file 6: Figure S2).

Although all beewolf-associated symbionts were originally assigned to biovars of the same species ‘*Ca.* S. philanthi’ [21], the morphology of even closely related biovars growing on the same medium varied strongly (Figure 4). On Grace’s medium, bacteria from the clade ‘*S. philanthi*’ formed pigmented (yellow or beige) opaque colonies of round or irregular form, flat or gibbous with wave, broken or smooth border, and the surface varied from matte to slightly shiny, from smooth to rimmed and rugose. Only biovar ‘*multimaculatus’*, when grown on the Grace’s insect medium, formed white colonies with well-developed aerial mycelium typical for *Streptomyces* (Figure 4).

Since all isolates were obtained on rich medium (supplemented with the full set of amino acids) imitating insect hemolymph, the next step was to assess the nutrient requirements of the isolated biovars by testing whether they could grow on media containing either an organic (peptones) or inorganic source of nitrogen (ammonium). Surprisingly, the majority of isolated biovars could grow (with different efficiencies) on the media containing peptones as the nitrogen source; only bacteria from the biovars ‘*loefflingi*’, ‘*triangulum diadema*’ and ‘*triangulum*’ (three Eurasian/African species taxa closely related to each other in both host and bacterial phylogenies) required medium imitating insect hemolymph (Figure 4). However, inorganic nitrogen was less suitable for supporting the growth of ‘*S. philanthi*’ strains: Only 11 out of 15 biovars isolated from North American *Philanthus* species as well as the symbiont of *Philanthinus quattuordecimpunctatus* were able to utilize ammonium as nitrogen source, but none of the isolates from European or African *Philanthus* or the South American *Trachypus* host species (Figure 4). Thus, the nitrogen assimilation pattern correlated strongly with geography and phylogeny of the hosts (Figure 4). The ability to assimilate inorganic nitrogen was also observed for all free-living species of the genus *Streptomyces* (*S. coelicolor*, *S. griseus*, *S. mobaraensis*, *S. avermitilis*, *S. cattleya*, *S. odorifer*, *S. viridochromogenes* and *S. antibioticus*) used for comparison in this work (Additional file 7: Figure S3). These bacteria were also growing on R2A and Grace’s media (data not shown). Interestingly, on R2A and on the medium containing ammonium, colonies with fuzzy surface formed by aerial mycelium, typical for free-living members of the genus *Streptomyces* and related Actinobacteria, were observed for the symbionts isolated from some North American *Philanthus* species (data not shown).

In order to gain more insight into physiological differences among symbiont biovars, resistance assays were performed with eight different antibiotics representing five major groups. The results revealed that antibiotic resistance of the isolated biovars also correlated with the host phylogeny. The biovars hosted by North American *Philanthus* as well as by *Philanthinus* were commonly antibiotic-resistant, especially to streptomycin, ampicillin and chloramphenicol (Table 1). By contrast, bacteria isolated from African and Eurasian *Philanthus* or South American *Trachypus* hosts were typically sensitive to the antibiotics applied: among these seven biovars, only three showed antibiotic resistance to streptomycin and just one to chloramphenicol. Generally, the isolated ‘*S. philanthi*’ biovars showed the highest sensitivity to rifampicin and tetracycline (Table 1).

**Table 1:** **Antibiotic resistance of ‘*S. philanthi*’ isolates**

**Geographic origin**	**Host species**	**Strain**				**Antibiotics**				
			**Aminoglucosides**			**Beta-lactam**		**Others**		
			**Sm**	**Km**	**Gm**	**Amp**	**PnG**	**Tet**	**Cm**	**Rif**
South America	*Trachypus elongatus*	elo 217	+	-	-	-	-	-	-	-
	*Trachypus flavidus*	fla 197	+	-	-	-	-	-	+	-
Eurasia/Africa	*Philanthus triangulum*	tri 23Af2	-	-	-	-	-	-	-	-
	*Philanthus triangulum diadema*	trd 176-2	-	-	-	-	-	-	-	-
Africa	*Philanthus histrio*	his 161	-	-	-	-	-	-	-	-
	*Philanthus loefflingi*	loe 163-1	-	-	-	-	-	-	-	-
	*Philanthus fuscipennis*	fus 137-1	+	-	-	-	-	-	-	-
North America	*Philanthus albopilosus*	alb 539-2	+	-	-	+	-	-	+	-
	*Philanthus barbiger*	bar 543-1	+	+	+	+	+	-	-	-
	*Philanthus bicinctus*	bic 628-2	+	-	-	+	+	-	+	−/+
	*Philanthus bilunatus*	bil 473-1	+	-	-	+	-	-	+	-
	*Philanthus crabroniformis*	cra 626-1	+	-	-	-	-	-	+	-
	*Philanthus gibbosus*	gib 464-1	+	+	+	-	-	-	+	-
	*Philanthus multimaculatus*	mul 415-1	+	+	+	+	-	−/+	+	−/+
	*Philanthus pacificus*	pac 380-2	+	-	-	-	-	-	+	-
	*Philanthus parkeri*	par 586-1	+	+	+	+	+	-	+	-
	*Philanthus politus*	pol 510-1	+	+	-	+	-	-	+	-
	*Philanthus psyche*	psy 534-1	+	-	-	+	+	−/+	-	-
	*Philanthus pulcher*	pul 394-1	+	+	-	-	-	-	+	-
	*Philanthus ventilabris*	ven 580-1	-	-	-	+	-	-	-	-
	*Philanthus zebratus*	zeb 627-1	+	+	+	+	+	-	+	-
Eurasia/Africa	*Philanthinus quattuordecimpunctatus*	qua 16-1	+	-	-	+	+	-	+	-

(+) indicates antibiotic resistance, (−) indicates antibiotic sensitivity.

(*Sm*) Streptomycin; (*Km*) Kanamycin; (*Gm*) Gentamycin; (*Amp*) Ampicillin; (*PnG*) Penicillin G; (*Tet*) Tetracycline; (*Cm*) Chloramphenicol; (*Rif*) Rifampicin.

Overall, the assessed physiological characteristics strongly varied across the monophyletic clade of *Streptomyces* symbionts, with the strains isolated from Eurasian/African *Philanthus* species showing the lowest metabolic versatility, followed by the South American *Trachypus*, while *Philanthinus* and the North American *Philanthus* species harboured symbionts that were more flexible in terms of nitrogen assimilation and antibiotic resistance.

### Diversity of symbiont strains within individual beewolf antennae

Since populations of symbiotic *Streptomyces* suffer significant bottlenecks during vertical transmission [26], genetic diversity within individual antennae could be expected to be low. However, recent phylogenetic analyses provided evidence for relatively frequent horizontal symbiont exchange among host species, raising the question whether individual antennae may in fact simultaneously harbour different bacterial lineages. Therefore, we set out to assess the diversity of symbionts growing within the same antenna.

For this analysis we used the antennae of two *P. multimaculatus* and one *P. psyche* specimen for the isolation of individual symbiont micro-colonies. These biovars were selected because in liquid medium they formed small (about 1 mm), compact, well-separated colonies. 24 individual colonies of each strain were harvested from the original enrichments and subjected to sequence analysis of the *gyrB* gene fragment, which provides higher phylogenetic resolution than the 16S rRNA gene. Perhaps due to different cell wall thickness, colony PCR and further sequence analysis succeeded with different efficiencies: 21 and 18 high quality sequences were obtained from the two ‘*multimaculatus*’ specimens (samples 570 and 571, respectively), but only six sequences from the ‘*psyche*’ biovar. Sequence analysis of *gyrB* revealed no heterogeneity among the analyzed isolates within each host individual, suggesting low levels of (micro) diversity or even clonality of the symbionts in individual beewolf antennae.

### Opportunistic bacteria

Beewolf antennae are constantly exposed to the environment, and non-specific bacteria are potentially able to colonize the gland reservoirs, especially in cases where the host fails to acquire its specific symbionts [28]. These bacteria, not belonging to the clade ‘*S. philanthi*’, were considered opportunistic, because no evidence for their contribution to the host’s protection against pathogens has been provided so far. Culturable forms of opportunistic bacteria were analyzed (i) from females’ antennal samples of different host species, and (ii) from European beewolf males’ antennae used as a reference for environmental contamination, because they do not contain antennal gland reservoirs.

**Figure 5:**
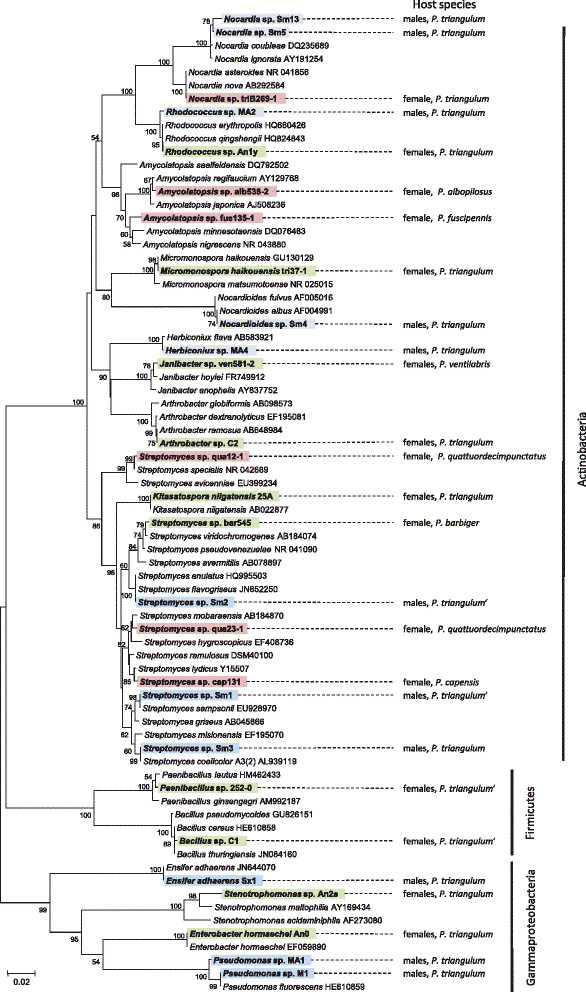
**Phylogenetic tree of opportunistic bacteria isolated from different beewolf samples.** Low-abundance bacteria (first dilution) were isolated from (i) males’ antennae (blue boxes) and (ii) females’ antennae (green boxes); high-abundance bacteria (10^2^-10^4^ CFU/sample) isolated from females’ antennae are shown in red boxes. The tree was reconstructed based on an alignment of 725 bp of 16S rRNA genes using Neighbour-Joining within MEGA version 5. Numbers at nodes indicate bootstrap values greater than 50%.

Based on their abundance, opportunistic bacteria isolated from both males’ and females’ antennae could be separated into two groups: all Gammaproteobacteria, Firmicutes, and Actinobacteria from males and some from females were isolated in low CFU counts (Figure 5). These bacteria were considered casual environmental contamination, probably of the antennal outer surface. The second group included highly abundant bacteria (10^2^-10^4^ CFU/sample), which were only isolated from females of different species and geographic origin; this group encompassed exclusively filamentous Actinobacteria (genera *Streptomyces*, *Amycolatopsis*, and *Nocardia*) (Figure 5). It seems likely that in those samples, the original symbiont from the clade ‘*S. philanthi*’ was replaced with other Actinobacteria in the antennal gland reservoirs, as has also been observed occasionally with molecular methods [28]. All these latter isolates were able to use ammonium as nitrogen source (data not shown).

## Discussion

In the present study, we report on the isolation of 22 wasp-associated ‘*S. philanthi*’ biovars in pure culture. Comparative physiological analyses provide insight into divergent metabolic capabilities in the monophyletic clade of symbiotic *Streptomyces*. Due to the difficulties in axenic cultivation of bacterial symbionts tightly associated with insect hosts, analyses of most symbiotic bacteria are confined to the *in silico* reconstruction of metabolic pathways from genomic or transcriptomic data. However, experiments on pure bacterial cultures can deliver direct evidence for the physiological consequences of co-evolution with the host and also provide the opportunity to test hypotheses on the symbionts’ physiology by genetic manipulation of the bacteria. Nevertheless, cultivation-based analyses also have important limitations: Since the conditions used for *in vitro* cultivation likely differ from those *in vivo*, the obtained results may not be representative of the natural situation.

Typically, bacteria of the genus *Streptomyces* possess large genomes (up to 11.9 Mb [32]) and therefore, they are capable of utilization and *de novo* biosynthesis of a large diversity of compounds. Concordantly, these bacteria can usually grow on simple mineral media with any one of a range of different carbon and nitrogen sources. However, ‘*S. philanthi*’ biovars isolated from the host genus *Trachypus*, and from African/Eurasian and some North American *Philanthus* species (*P. ventilabris*, *P. bilunatus*, *P. multimaculatus* and *P. pulcher*) were unable to assimilate inorganic nitrogen (which free-living streptomycetes typically can) and needed peptides or even more complex media imitating insect hemolymph (biovars ‘*triangulum*’, ‘*triangulum diadema*’ and ‘*loefflingi*’). Additionally, they were sensitive to a broad range of antibiotics. These characteristics suggest that their co-evolution with wasps resulted in decreased metabolic versatility, probably caused by genome erosion; this phenomenon is well known for symbiotic bacteria tightly associated with their hosts [29],[30].

Considering the monophyly of the ‘*S. philanthi*’ clade and the observation that they populate phylogenetically and ecologically similar host taxa, we expected that different ‘*S. philanthi*’ biovars share similar physiological characteristics. In contrast to that anticipation, however, isolated ‘*S. philanthi*’ strains showed broad diversity in morphology and physiology. While the observed physiological patterns also showed some congruency with the symbiont phylogenetic relationships, the host phylogeny appeared to be a much better predictor of symbiont physiology, specifically considering the group requiring hemolymph-imitating nutrient medium (symbionts of *P. triangulum, P. triangulum diadema,* and *P. loefflingi*), as well as the physiologically similar *Trachypus* symbionts (biovars ‘*elongatus’* and ‘*flavidus’*), which both turned out as monophyletic in the host but not symbiont phylogeny (Figure 4). Thus, the environment provided by the host in the antennal gland reservoirs seems to be an important factor shaping the evolutionary fate of the symbionts.

The differences in metabolic versatility across symbiont strains may reflect different stages of genome erosion. In intracellular insect symbionts, degenerative genome evolution of bacterial symbionts commonly proceeds comparatively quickly within the first phase of intimate associations, followed by genomic stasis [33],[34]. In beewolves, however, our results and previous co-phylogenetic analyses with fossil calibration suggest that the symbionts’ loss of metabolic capabilities has started long after the origin of the symbiosis in the late Cretacious [28] and proceeded independently in particular clades, as exemplified by the loss of metabolic capabilities and antibiotic resistance in the symbionts of defined host lineages (Figure 4). Preliminary data from ongoing genome sequencing projects of four ‘*S. philanthi*’ biovars support the hypothesis of independent genome evolution in different symbiont lineages (Nechitaylo et al., unpubl. data).

**Figure 6:**
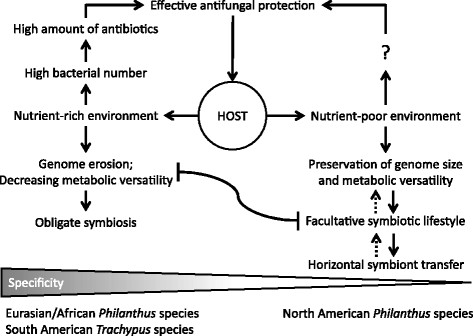
**Scheme of putative host-driven evolution within the monophyletic clade ‘*S. philanthi*’.**

At present, we can only speculate about the mechanistic basis of the host influence on symbiont physiology. A plausible scenario, however, is that the amount, complexity, and reliability of nutrients provided to the symbionts can affect the symbionts’ evolutionary fate by relaxing or increasing selective pressures on maintaining metabolic versatility. Under this scenario, a nutrient-rich and stable environment provided by the host sustains genome erosion in the symbiotic bacteria, leading to metabolic dependency and high host specificity (Figure 6). Despite the higher costs, providing a rich environment could be beneficial to the host by stimulating bacterial growth and increasing the number of bacterial cells applied onto the cocoon, which in turn leads to high antibiotic production and an effective symbiont-mediated host protection [35]. Simultaneously, a rich environment could allow for selection of the best symbionts by ‘screening’ through increased competition, with the most competitive and best-defended strain winning out [36],[37]. By contrast, a nutrient-poor environment (lower amount, diversity, and/or reliability of nutrients) would be less costly to the host and prevent genome erosion in the bacterial symbionts. The high metabolic versatility would enable the bacteria to persist as free-living forms and provide the opportunity for host switching by horizontal transfer (Figure 6). Interestingly, different symbiont strains across individuals of the same host species have so far only been detected for North American *Philanthus* species ([28], this study: biovar ‘*albopilosus’* strains alb539-2), suggesting that horizontal transfer of symbionts is indeed more common among these physiologically versatile strains than across species in the metabolically more restricted South American and Eurasian/African clades. Such horizontal transfer could occur in populations of sympatric host species through interspecific predation or by the acquisition of symbionts from the soil in reused or closely associated brood chambers (Figure 6).

Acquisition of symbionts occurs shortly before or during emergence of the adult female beewolf from the cocoon, and only few bacterial cells are taken up into the antennal gland reservoirs [26]. The strong bottleneck effect likely contributes to the low genetic diversity we observed within the antennae of individual beewolves, as well as across host individuals of the same species (see also [28]). While the genetic homogeneity of the symbionts reduces competition and conflict in the symbiosis, it also compromises the symbionts’ ability to adapt to changing environmental conditions [38]. Furthermore, the uptake of low numbers of symbiont cells from the cocoon surface may entail the risk of taking up non-symbiotic bacteria into the antennae.

Accordingly, opportunistic bacteria were occasionally isolated from beewolf antennae, indicating that replacement of the beewolf symbionts with opportunistic bacteria can indeed occur (see also [28]). Interestingly, however, our results suggest that only filamentous Actinobacteria (genera *Streptomyces*, *Amycolatopsis* and *Nocardia*) can reach high densities and persist in the antennal gland reservoirs, whereas other bacteria probably contaminate the antennal surface in low abundance, but do not invade the reservoirs. Thus, the host apparently provides a selective environment that acts as a first ‘screening’ mechanism to prevent the growth of many opportunistic, and possibly pathogenic, bacteria [36]. As a second step to ensure partner specificity, the host selectively blocks application of opportunistic Actinobacteria from the gland reservoirs into the brood cells, thereby effectively disrupting the vertical transmission route [28]. Despite the opportunity for acquisition of opportunistic bacteria, the combination of these two different layers of symbiont selection seem to efficiently ensure specificity in the association over long evolutionary timescales, as reflected in the monophyly of the beewolf symbiont clade.

## Conclusion

The successful *in vitro* cultivation and characterization of multiple defensive symbiont strains of beewolves provided valuable insight into the symbionts’ physiology and revealed an unexpected morphological and physiological diversity that may reflect a ‘snapshot’ of ongoing evolution towards a tight association with the wasps. We hypothesize that the selective host environment plays an important role in shaping degenerative metabolic evolution in its native symbionts and also acts as a ‘screening’ barrier to prevent colonization by potentially pathogenic microorganisms.

## Methods

### Beewolf antennae sampling

Beewolf females were taken from a laboratory colony (*Philanthus triangulum*, originally collected in Berlin, Germany) or collected in their natural habitats in Berlin (Germany), Turkey (Erzurum), South Africa (Eastern and Western Cape provinces), USA (Utah and New Hampshire) and Brazil (São Paulo province) (see Additional file 3: Table S3). One antenna from each caught female was cut and stored air-dried in sterile Eppendorf tubes at room temperature or in the fridge (when available) for up to two weeks.

### Isolation of bacterial symbionts

Beewolf antennal specimens were crushed in 1.5 ml sterile tubes (Eppendorf) containing 50–150 μl liquid nutrient medium using sterile 1 ml pipette tips, in order to release symbiotic bacteria from the antennal glands. After that, the antennal samples were transferred into 24-well plates with liquid media (0.5 ml/well) and serially diluted up to 10^−2^ – 10^−3^ in order to avoid overgrowth of possible contamination. The plate was sealed with parafilm or put into a disposable plastic bag for incubation at 27-30°C. Initially, three different media were designed (Additional file 1: Table S1) and applied to isolate ‘*Ca*. Streptomyces philanthi biovar triangulum’. Subsequently, the following ready-to-use media were also used: media for different insect cell lines (Grace’s, TC-100, Schneider’s *Drosophila* medium (Sigma and Gibco), and SF900-II (Gibco)), media for mammalian cell lines (DMEM, CMRL, M199 and RPMI 1640 (all Gibco)) (Additional file 2: Table S2), and standard bacterial media (R2A and Actinomycete Isolation Agar (all Sigma)). Later, Grace’s medium with 10% fetal bovine serum, FBS, (Lonza) was used for the isolation of other biovars. Since streptomycetes growing in liquid medium form compact colonies, the following strategy was applied to isolate a pure culture: single colonies were transferred into individual wells of 24-well plate containing 500 μl fresh medium and were disrupted by pipetting. After that, bacteria were incubated again until new micro-colonies appeared and the procedure was repeated three times. Finally, bacterial biomass was stored at −80°C with glycerol (15-20%) added to liquid medium. Bacterial isolates were named with the first three letters of the host species name, plus the running number for the host specimen according to our internal collection, and a number referring to the replicate isolate (e.g. alb539-2 refers to isolate 2 of the *Philanthus albopilosus* specimen no. 539).

### DNA extraction, PCR amplification, and identification of isolates

Bacteria grown in appropriate liquid medium were collected in 1.5 ml tubes by centrifugation at 5000 × *g* for 1 min at room temperature and washed twice with sterile PBS (137 mM NaCl; 2.7 mM KCl; 10 mM Na_2_HPO_4_; 2 mM KH_2_PO_4_). The bacterial biomass was lysed as described elsewhere [39]: briefly, the biomass was resuspended in 500 μl TE25S buffer (25 mM Tris (pH 8.0), 25 mM EDTA (pH 8.0), 0.3 M sucrose) with lysozyme (2 mg/ml) and incubated at 37°C for 1 h. Afterwards, 50 μl proteinase K (20 mg/ml) and 30 μl SDS (10%) were added, mixed and the samples were incubated at 55°C with agitation for 20 min. 100–200 μl Protein Precipitation Solution (Qiagen) was added to the transparent lysate, which was then thoroughly mixed and centrifuged at >16,000 × *g* for 10 min at 4°C to sediment proteins. The supernatant was transferred into a fresh tube, and an equal volume (i.e. 600–700 μl) of isopropanol was added; the solution was thoroughly mixed and the tube was incubated at −20°C for ≥30 min, followed by centrifugation at ≥16,000 × *g* for 10 min to sediment DNA. The DNA pellet was then washed twice with 500 μl EtOH (70%), air-dried, and resuspended in EB buffer.

Bacterial 16S rRNA gene fragments were amplified with the primers fD1 (5’-AGAGTTTGATCCTGGCTCAG-3’) and rP2 (5’-ACGGCTACCTTGTTACGACTT-3’); gyrase subunit A (*gyrA*) gene fragments were amplified with gyrA-5F (5’-AACCTGCTGGCCTTCCAG-3’) and gyrA-5R (5’-AACGCCCATGGTGTCACG-3’); gyrase subunit B (*gyrB*) gene fragments were amplified with primers gyrB-F1 (5’-GAGGTCGTGCTGACCGTGCTGCA-3’) and gyrB-R3 (5’-SAGCTTGACCGAGATGATCG-3’) [28]. PCR amplifications were performed on a Biometra® T-Gradient Thermocycler or on a VWR Gradient Thermocycler using *Taq* DNA polymerase (MBI Fermentas) according to the manufacturer’s protocol with annealing at 55°C, 60°C and 68°C (for 16S rRNA, *gyrA* and *gyrB*, respectively) for 40 sec. PCR products were purified with QIAquick PCR Purification Kit (Qiagen) and sequenced with primers fD1, rP2 and R1087 (5’-CTCGTTGCGGCACTTAACCC-3’), gyrA-5F, and gyrB-F1, respectively. Sequencing was done in the Department of Entomology at the Max Planck Institute for Chemical Ecology (Jena, Germany) or commercially by SEQLAB Sequence Laboratories (Göttingen, Germany). Bacterial sequences were deposited in the GenBank database under following accession numbers: KM035545 - KM035652 (16S rRNA genes), KM035653 - KM035673 (*gyrA* genes) and KM035674 - KM035755 (*gyrB* genes).

### Diversity of bacterial strains in individual beewolf antennae

Bacterial micro-colonies were isolated from individual antennae of two different *Philanthus multimaculatus* and one *Philanthus psyche* female with serial dilution in 24-well plates with liquid medium as described above. Individual micro-colonies were carefully transferred by pipette into 96-well PCR plates with 100 μl PCR lysis solution A without proteinase K (67 mM Tris–HCl (pH 8.8); 16.6 mM (NH_4_)_2_SO_4_; 6.7 mM MgCl_2_; 6.7 μM EDTA (pH 8.0); 1.7 μM SDS; 5 mM β-mercaptoethanol) [40]; samples were heated at 95°C for 5 min to destroy bacterial cells. Afterwards, *gyrB* gene fragments were amplified, purified and sequenced as described above. Obtained sequences were aligned and manually curated using Geneious software version 6.0.5 (Biomatters Ltd., http://www.geneious.com/).

### Phylogenetic analysis

16S rRNA, *gyrA* and *gyrB* gene sequences of isolated symbionts were aligned with those obtained from field-collected beewolves as well as representative outgroup sequences of free-living *Streptomyces* and other actinomycete strains (Additional file 4: Table S4). Alignments of individual genes were concatenated for phylogenetic analyses. Approximately-maximum-likelihood trees were reconstructed with FastTree 2.1 using the GTR model, with local support values estimated by the Shimodaira-Hasegawa test based on 1,000 resamplings without re-optimizing the branch lengths for the resampled alignments [41].

Bayesian inferences were run with MrBayes 3.1.2 [42]–[44], with the different genes defined as separate partitions in the concatenated alignment. The searches were conducted under the GTR + I + G model, with 4,000,000 generations per analysis. Trees were sampled every 1,000 generations. We confirmed that the standard deviation of split frequencies was consistently lower than 0.01, and a “burnin” of 25% was used, i.e. the first 25% of the sampled trees were discarded. We computed a 50% majority rule consensus tree with posterior probability values for every node.

For reconstructing the host phylogeny, sequences of five nuclear [28S rRNA, wingless (wnt), long-wavelength rhodopsin (*lwrh*), arginine kinase (*argK*), and elongation factor 1α (*ef1a*)] and one mitochondrial gene [cytochrome oxidase (*coxI*)] for 22 Philanthini of three genera (*Philanthus, Trachypus, Philanthinus*) were obtained from the NCBI database [for accession numbers, see ref. 28]. A phylogenetic tree was reconstructed using the GTR model in FastTree 2.1 [41].

Phylogenetic analysis of 16S rRNA gene fragments from opportunistic bacteria was conducted using MEGA version 5 [45].

### Fluorescence *in situ* hybridization (FISH)

Bacteria grown in liquid M552 medium or bacteria directly from antennal samples were fixed in 4% formaldehyde overnight at 4°C, washed twice with ice-cold PBS and used for fluorescence *in situ* hybridization (FISH) as previously described [21]. The samples were dehydrated in a graded ethanol series and mounted on microscope slides coated with poly-L-lysine (Kindler, Freiburg, Germany). FISH was done with the ‘*Ca*. Streptomyces philanthi’-specific oligonucleotide probe Cy3-SPT177 [21] or the general eubacterial probe Cy3-EUB338 [46]. Additionally, bacterial DNA was stained unspecifically with DAPI (4’, 6-diamidino-2-phenylindole). Bacteria were visualized using an AxioImager.Z1 microscope (Zeiss).

### Analysis of the symbionts’ nutritional requirements

In order to assess nutrient requirements, bacteria grown in liquid Grace’s medium with 10% FBS were seeded onto R2A agar (Sigma) or onto agarified Grace’s medium containing inorganic salts, vitamins and carbon sources (sucrose, glucose and fructose), as well as one of two different nitrogen sources: (i) peptones from casein (Serva) and tryptone (AppliChem) 5 g/L each, or (ii) ammonium chloride 1 g/L. Bacteria were incubated in 24-well plates as described above.

### Antibiotic resistance assays

In order to analyze antibiotic resistance, bacteria were grown in liquid Grace’s medium supplemented with the following antibiotics (final concentrations): ampicillin (100 μg/ml), penicillin G (100 μg/ml), chloramphenicol (25 μg/ml), streptomycin (50 μg/ml), gentamycin (50 μg/ml), kanamycin (50 μg/ml), rifampicin (50 μg/ml), tetracycline (15 μg/ml). Bacterial growth was assessed visually after two weeks of incubation at 28°C as described above, in comparison with control samples grown without antibiotics.

### Scanning electron microscopy (SEM)

For the SEM analysis, bacteria were grown as colonies on agarified Grace’s medium at 28°C for 1 month and then incubated at 10-14°C for an additional three weeks. Agar blocks with bacterial colonies were cut out, fixed overnight with 2,5% glutaraldehyde in sodium cacodylate buffer (0.1 M, pH 7.0) and were dehydrated with ethanol in serially increased concentration, followed by critical point drying in a Leica EM CPD300 Automated Critical Point Dryer (Leica, Wetzlar, Germany). The samples were sputter coated with gold (layer 30 nm) in a BAL-TEC SCD005 Sputter Coater (BAL-TEC, Liechtenstein) and analyzed at different magnifications with a LEO 1530 Gemini field emission scanning electron microscope (Zeiss, Oberkochen, Germany) at 5 kV acceleration voltage and a working distance of 5 mm using an inlense secondary electron detector.

### Amplified Fragment Length Polymorphism (AFLP)

Genomic DNA from individual symbiont strains was used for AFLP as described by [47]. Briefly, DNA was digested with the two restriction enzymes *Apa*I (4U) and *Taq*I (4U), and *Apa*I and *Taq*I adapters were added (Additional file 8: Table S5). After pre-amplifying the ligation product, selective amplifications were conducted using the two differently labeled primers TaqI-G (IRDye 700) and TaqI-C (IRDye 800) in combination with one out of ten *Apa*I primers with two selective nucleotides (see Additional file 8: Table S5). Amplified products were separated based on size with a LI-COR DNA Analyzer 4300. A formamide-dye stop solution was added to the AFLP reactions, and samples were heat-denatured before electrophoresis. For separation, a 6.5% polyacrylamide gel was used, and a labeled size standard was loaded at each end. Gels were run for 2.5 h and subsequently scored using the software AFLP-Quantar™ Pro 1.0 (KeyGene Products, Wageningen, The Netherlands). Scoring results of 202 AFLP markers were converted into ‘pseudo-sequences’ (with presence = ‘A’, absence = ‘T’, and unknown = ‘N’), imported into MEGA5.01 [45], and used to construct a neighbour-joining phylogeny including 100 replicates for bootstrap analysis.

## Competing interests

The authors declare that they have no competing interests.

## Authors’ contributions

TYN and MK participated in the isolation/characterization of bacterial symbionts and in the design of the study. MW performed the electron microscopy. All authors read and approved the final manuscript.

## Additional files

## Supplementary Material

Additional file 1: Table S1. Composed media recipes. Click here for file

Additional file 2: Table S2. Composition of commercial cell line media used in this work (amounts in mg/L). Click here for file

Additional file 3: Table S3. Number of ‘*S. philanthi*’ CFUs isolated from different females’ antennal samples. Click here for file

Additional file 4: Table S4. Accession numbers of actinobacterial sequences included in the phylogenetic analyses shown in Figure 3. Click here for file

Additional file 5: Figure S1. AFLP-based phylogenetic analysis of cultured ‘*S. philanthi’* biovars. Click here for file

Additional file 6: Figure S2. Polymorphism of ‘*S. philanthi*’ biovars ‘*elongatus*’ and ‘*loefflingi*’. Click here for file

Additional file 7: Figure S3. Free-living bacteria growing on the solid modified Grace’s medium with ammonium as the only nitrogen source. Click here for file

Additional file 8: Table S5. Primers and adapters used for generation of AFLP markers. Click here for file

## References

[B1] Moran NA Symbiosis Curr Biol 2006 16 R866 R871 10.1016/j.cub.2006.09.019 17055966

[B2] Feldhaar H Bacterial symbionts as mediators of ecologically important traits of insect hosts Ecol Entomol 2011 36 533 543 10.1111/j.1365-2311.2011.01318.x

[B3] Pontes MH Dale C Culture and manipulation of insect facultative symbionts Trends Microbiol 2006 14 406 412 10.1016/j.tim.2006.07.004 16875825

[B4] Kim JK Won YJ Nikoh N Nakayama H Han SH Kikuchi Y Rhee YH Park HY Kwon JY Kurokawa K Dohmae N Fukatsu T Lee BL Polyester synthesis genes associated with stress resistance are involved in an insect-bacterium symbiosis Proc Natl Acad Sci U S A 2013 110 E2381 E2389 10.1073/pnas.1303228110 23757494 PMC3696747

[B5] Kim JK, Lee HJ, Kikuchi Y, Kitagawa W, Nikoh N, Fukatsu T, Lee BL: **Bacterial cell wall synthesis gene uppP is required for*****Burkholderia*****colonization of the stinkbug gut.** *Appl Environ Microbiol* 2013, **79:**4879–4886. 10.1128/AEM.01269-13 PMC3754700 23747704

[B6] Dale C Beeton M Harbison C Jones T Pontes M Isolation, pure culture, and characterization of “Candidatus Arsenophonus arthropodicus”, an intracellular secondary endosymbiont from the hippoboscid louse fly Pseudolynchia canariensis Appl Environ Microbiol 2006 72 2997 3004 10.1128/AEM.72.4.2997-3004.2006 16598007 PMC1449044

[B7] Dulla GFJ Go RA Stahl DA Davidson SK Verminephrobacter eiseniae type IV pili and flagella are required to colonize earthworm nephridia ISME J 2012 6 1166 1175 10.1038/ismej.2011.183 22170422 PMC3358029

[B8] Bourtzis K Miller TA Insect Symbiosis 2004 Taylor & Francis, Boca Raton, USA

[B9] Brownlie JC Johnson KN Symbiont-mediated protection in insect hosts Trends Microbiol 2009 17 348 354 10.1016/j.tim.2009.05.005 19660955

[B10] Kaltenpoth M Engl T Defensive microbial symbionts in Hymenoptera Functional Ecol 2013 28 315 327 10.1111/1365-2435.12089

[B11] Seipke RF Kaltenpoth M Hutchings MI Streptomyces as symbionts: an emerging and widespread theme? FEMS Microbiol Rev 2011 36 862 876 10.1111/j.1574-6976.2011.00313.x 22091965

[B12] Kaltenpoth M Actinobacteria as mutualists: general healthcare for insects? Trends Microbiol 2009 17 529 535 10.1016/j.tim.2009.09.006 19853457

[B13] Currie CR Scott JA Summerbell RC Malloch D Fungus-growing ants use antibiotic-producing bacteria to control garden parasites Nature 1999 398 701 704 10.1038/19519

[B14] Haeder S, Wirth R, Herz H, Spiteller D: **Candicidin-producing*****Streptomyces*****support leaf-cutting ants to protect their fungus garden against the pathogenic fungus*****Escovopsis*****.** *Proc Natl Acad Sci U S A* 2009, **106:**4742–4746. 10.1073/pnas.0812082106 PMC2660719 19270078

[B15] Barke J, Seipke RF, Gruschow S, Heavens D, Drou N, Bibb MJ, Goss RJ, Yu DW, Hutchings MI: **A mixed community of actinomycetes produce multiple antibiotics for the fungus farming ant*****Acromyrmex octospinosus*****.** *BMC Biol* 2010, **8:**109. 10.1186/1741-7007-8-109 PMC2942817 20796277

[B16] Scott JJ Oh DC Yuceer MC Klepzig KD Clardy J Currie CR Bacterial protection of beetle-fungus mutualism Science 2008 322 63 10.1126/science.1160423 18832638 PMC2761720

[B17] Kaltenpoth M Gottler W Herzner G Strohm E Symbiotic bacteria protect wasp larvae from fungal infestation Curr Biol 2005 15 475 479 10.1016/j.cub.2004.12.084 15753044

[B18] Poulsen M Oh DC Clardy J Currie CR Chemical analyses of wasp-associated streptomyces bacteria reveal a prolific potential for natural products discovery PLoS One 2011 6 e16763 10.1371/journal.pone.0016763 21364940 PMC3043073

[B19] Le Roes-Hill M Rohland J Burton S Actinobacteria isolated from termite guts as a source of novel oxidative enzymes Antonie Van Leeuwenhoek Int J Gen Mol Microbiol 2011 100 589 605 10.1007/s10482-011-9614-x 21720857

[B20] Seipke RF, Barke J, Ruiz-Gonzalez MX, Orivel J, Yu DW, Hutchings MI: **Fungus-growing*****Allomerus*****ants are associated with antibiotic-producing actinobacteria.** *Antonie Van Leeuwenhoek* 2012, **101:**443–447. 10.1007/s10482-011-9621-y 21748399

[B21] Kaltenpoth M, Goettler W, Dale C, Stubblefield JW, Herzner G, Roeser-Mueller K, Strohm E: ***‘Candidatus*****Streptomyces philanthi’, an endosymbiotic streptomycete in the antennae of*****Philanthus*****digger wasps.** *Int J Syst Evol Microbiol* 2006, **56:**1403–1411. 10.1099/ijs.0.64117-0 16738121

[B22] Kaltenpoth M, Yildirim E, Gurbuz MF, Herzner G, Strohm E: **Refining the roots of the beewolf-*****Streptomyces*****symbiosis: antennal symbionts in the rare genus*****Philanthinus*****(Hymenoptera, Crabronidae).** *Appl Environ Microbiol* 2012, **78:**822–827. 10.1128/AEM.06809-11 PMC3264120 22113914

[B23] Kaltenpoth M, Schmitt T, Strohm E: **Hydrocarbons in the antennal gland secretion of female European beewolves,*****Philanthus triangulum*****(Hymenoptera, Crabronidae).** *Chemoecol* 2009, **19:**219–225.

[B24] Goettler W, Kaltenpoth M, Herzner G, Strohm E: **Morphology and ultrastructure of a bacteria cultivation organ: the antennal glands of female European beewolves,*****Philanthus triangulum*****(Hymenoptera, Crabronidae).** *Arthropod Struct Dev* 2007, **36:**1–9. 10.1016/j.asd.2006.08.003 18089083

[B25] Kroiss J Kaltenpoth M Schneider B Schwinger MG Hertweck C Maddula RK Strohm E Svatos A Symbiotic Streptomycetes provide antibiotic combination prophylaxis for wasp offspring Nat Chem Biol 2010 6 261 263 20190763 10.1038/nchembio.331

[B26] Kaltenpoth M Goettler W Koehler S Strohm E Life cycle and population dynamics of a protective insect symbiont reveal severe bottlenecks during vertical transmission Evol Ecol 2010 24 463 477 10.1007/s10682-009-9319-z

[B27] Koehler S Doubsky J Kaltenpoth M Dynamics of symbiont-mediated antibiotic production reveal efficient long-term protection for beewolf offspring Front Zool 2013 10 3 10.1186/1742-9994-10-3 23369509 PMC3599432

[B28] Kaltenpoth M Roeser-Mueller M Koehler S Peterson A Nechitaylo T Stubblefield JW Herzner G Seger J Strohm E Partner choice and fidelity stabilize co-evolution in a Cretaceous-age defensive symbiosis Proc Natl Acad Sci U S A 2014 111 6359 6364 10.1073/pnas.1400457111 24733936 PMC4035988

[B29] McCutcheon JP Moran NA Extreme genome reduction in symbiotic bacteria Nat Rev Microbiol 2012 10 13 26 22064560 10.1038/nrmicro2670

[B30] Ochman H Genomes on the shrink Proc Natl Acad Sci U S A 2005 102 11959 11960 10.1073/pnas.0505863102 16105941 PMC1189353

[B31] Murray RG Stackebrandt E Taxonomic note: implementation of the provisional status Candidatus for incompletely described procaryotes Int J Syst Bacteriol 1995 45 186 187 10.1099/00207713-45-1-186 7857801

[B32] Wang XJ, Yan YJ, Zhang B, An J, Wang JJ, Tian J, Jiang L, Chen YH, Huang SX, Yin M, Zhang J, Gao AL, Liu CX, Zhu ZX, Xiang WS: **Genome sequence of the milbemycin-producing bacterium*****Streptomyces bingchenggensis*****.** *J Bacteriol* 2010, **192:**4526–4527. 10.1128/JB.00596-10 PMC2937363 20581206

[B33] Tamas I Klasson L Canback B Naslund AK Eriksson AS Wernegreen JJ Sandstrom JP Moran NA Andersson SG 50 million years of genomic stasis in endosymbiotic bacteria Science 2002 296 2376 2379 10.1126/science.1071278 12089438

[B34] McCutcheon JP Moran NA Functional convergence in reduced genomes of bacterial symbionts spanning 200 My of evolution Genome Biol Evol 2010 2 708 718 20829280 10.1093/gbe/evq055 PMC2953269

[B35] Koehler S Kaltenpoth M Maternal and environmental effects on symbiont-mediated antimicrobial defense J Chem Ecol 2013 39 978 988 10.1007/s10886-013-0304-1 23779268

[B36] Scheuring I Yu DW How to assemble a beneficial microbiome in three easy steps Ecol Lett 2012 15 1300 1307 10.1111/j.1461-0248.2012.01853.x 22913725 PMC3507015

[B37] Archetti M Scheuring I Hoffman M Frederickson ME Pierce NE Yu DW Economic game theory for mutualism and cooperation Ecol Lett 2011 14 1300 1312 10.1111/j.1461-0248.2011.01697.x 22011186

[B38] Sachs JL Skophammer RG Regus JU Evolutionary transitions in bacterial symbiosis Proc Natl Acad Sci U S A 2011 108 Suppl 2 10800 10807 10.1073/pnas.1100304108 21690339 PMC3131820

[B39] Kieser T Bibb MJ Buttner MJ Chater KF Hopwood DA Practical streptomyces genetics 2000 John Innes Foundation, Norwich, England

[B40] Sambrook J Russell D Molecular Cloning: A Laboratory Manual 2001 Cold Spring Harbor Laboratory Press, New York, USA

[B41] Price MN Dehal PS Arkin AP FastTree 2–approximately maximum-likelihood trees for large alignments PLoS One 2010 5 e9490 10.1371/journal.pone.0009490 20224823 PMC2835736

[B42] Huelsenbeck JP Ronquist F MRBAYES: Bayesian inference of phylogenetic trees Bioinform 2001 17 754 755 10.1093/bioinformatics/17.8.754 11524383

[B43] Huelsenbeck JP Ronquist F Nielsen R Bollback JP Bayesian inference of phylogeny and its impact on evolutionary biology Science 2001 294 2310 2314 10.1126/science.1065889 11743192

[B44] Ronquist F Huelsenbeck JP MrBayes 3: Bayesian phylogenetic inference under mixed models Bioinform 2003 19 1572 1574 10.1093/bioinformatics/btg180 12912839

[B45] Tamura K Peterson D Peterson N Stecher G Nei M Kumar S MEGA5: Molecular Evolutionary Genetics Analysis using maximum likelihood, evolutionary distance, and maximum parsimony methods Mol Biol Evol 2011 28 2731 2739 10.1093/molbev/msr121 21546353 PMC3203626

[B46] Amann RI Binder BJ Olson RJ Chisholm SW Devereux R Stahl DA Combination of 16S rRNA-targeted oligonucleotide probes with flow cytometry for analyzing mixed microbial populations Appl Environ Microbiol 1990 56 1919 1925 2200342 10.1128/aem.56.6.1919-1925.1990 PMC184531

[B47] Jin QC Jin ZH Xu B Wang Q Lei YL Yao SJ Cen PL Genomic variability among high pristinamycin-producing recombinants of Streptomyces pristinaespiralis revealed by amplified fragment length polymorphism Biotechnol Lett 2008 30 1423 1429 10.1007/s10529-008-9701-x 18368295

