# Supplementary material for: Cultivation reveals physiological diversity among defensive ‘Streptomyces philanthi’ symbionts of beewolf digger wasps (Hymenoptera, Crabronidae)
Source: BMC Microbiol. 2014 Jul 29;14:202. doi: 10.1186/s12866-014-0202-x (PMC4236554; doi:10.1186/s12866-014-0202-x)
Supplement: Additional file 1: Table S1. — Composed media recipes. [file s12866-014-0202-x-S1.pdf]

Table S1. Composed media recipes.

**Component**	**Medium**		
	**M522**	**M252**	**M225**
Grace's medium	5*	2	2
DMEM	2	5	2
CMRL	2	2	5
Hank's Balanced Salt Solution (Sigma)	1	1	1
Insect Medium Supplement (Sigma)	1	1	1
Fetal Bovine Serum (Lonza)	1	1	1
pH	6.5-6.8	7.5-7.8	7.5-7.8

* - volume in ml
