# Supplementary material for: Cultivation reveals physiological diversity among defensive ‘Streptomyces philanthi’ symbionts of beewolf digger wasps (Hymenoptera, Crabronidae)
Source: BMC Microbiol. 2014 Jul 29;14:202. doi: 10.1186/s12866-014-0202-x (PMC4236554; doi:10.1186/s12866-014-0202-x)
Supplement: Additional file 2: Table S2. — Composition of commercial cell line media used in this work (amounts in mg/L). [file s12866-014-0202-x-S2.pdf]

Table S2. Composition of commercial cell line media used in this work (amounts in mg/L)

**Amino Acids**	**Grace's**	**TC-100**	**Schneider's**	**DMEM 21885†**	**CMRL**	**Medium 199**	**RPMI 1640**
Glycine	650	650	250	30	50	50	10
L-Alanine	225	225	-	862	10	25	-
L-Arginine hydrochloride	700	700	600	84	25	70	200
L-Asparagine	350	350	-	-	70	-	50
L-Aspartic acid	350	350	400	-	30	30	20
L-Cysteine 2HCl	-	-	-	-	-	0.1	-
L-Cystine	28.68	25	26.7	48	199.88	26	65
L-Glutamic Acid	600	600	800	-	20	75	20
L-Glutamine	600	600	1800	862	75	100	300
L-Histidine	2500	2500	400	42	20	21.88	15
L-Isoleucine	50	50	150	105	20	10	50
L-Hydroxyproline	-	-	-	-	-	40	20
L-Leucine	75	75	150	105	60	60	50
L-Lysine Hydrochloride	625	625	2060.8	146	70	70	40
L-Methionine	50	50	150	30	15	15	15
L-Phenylalanine	150	150	-	66	25	25	15
L-Proline	350	350	1700	-	40	40	20
L-Serine	550	550	250	42	25	25	30
L-Threonine	175	175	350	95	30	30	20
L-Tryptophan	100	100	100	16	10	10	5
L-Tyrosine disodium salt	62.14	72	720	72	40	58	29
L-Valine	100	100	300	94	25	25	20
beta-Alanine	200	-	500	-	-	-	
**Vitamins**							
Ascorbic Acid	-	-	-	-	50	0.05	-
Biotin	0.01	0.01	0.08	-	0.01	0.01	0.2
Choline chloride	0.2	0.2	1.60	4	0.5	0.5	3.0
D-Calcium pantothenate	0.02	0.02	0.16	4	0.01	0.01	0.25
Folic Acid	0.02	0.02	0.16	4	0.01	0.01	1.0
i-Inositol	0.02	-	0.16	7.2	0.05	0.05	35.0
Niacinamide	-	-	-	4	0.025	0.025	1.0
Nicotinic acid	0.02	0.02	0.16	-	0.025	0.025	-
Para-Aminobenzoic Acid	0.02	0.02	0.16	-	0.05	0.05	1.0
Pyridoxal hydrochloride	-	-	-	-	-	0.025	1.0
Pyridoxine HCl	0.02	0.02	0.16	4	0.025	0.025	-
Riboflavin	0.02	0.02	0.16	0.4	0.01	0.01	0.2
Thiamine hydrochloride	0.02	0.02	0.16	4	0.01	0.01	1.0
Vitamin A	-	-	-	-	-	0.1	-
Alpha-tocopherol Phosphate	-	-	-	-	-	0.01	-
Menadione	-	-	-	-	-	0.01	-
Calciferol	-	-	-	-	-	0.1	-
Cobalamin	-	-	-	-	-	-	0.0050
**Inorganic salts**							
$Fe(NO_3)_3 * 9H_2O$	-	-	-	0,1	-	0.7	-
$Ca(NO_3)_2 * 4H_2O$	-	-	-	-	-	-	100.0

$CaCl_2$	500	9966	-	264	264	200	-
$MgCl_2$	1070	1068	1807	-	-	-	-
$MgSO_4$	1358	1358	-	200	200	97.67	48.84
KCl	2800	2870	1600	400	400	400	400.0
KH_2PO_4	-	-	450	-	-	-	-
$NaHCO_3$	350	350	-	3700	2200	2200	2000.0
NaCl	-	-	2100	6400	6799	6800	6000.0
Na_2HPO_4	-	-	700	-	-	-	800.0
$NaH_2PO_4 * H_2O$	1013	876,9	-	154	158	140	-
**Reducing Agents**							
Glutathione, monosodium	-	-	-	-	10	0.05	1.0
**Carbon sources**							
D-Glucose (Dextrose)	700	1000	2000	1000	1000	1000	2000
D-Fructose	400	-	-	-	-	-	-
Sucrose	26680	-	-	-	-	-	-
Ribose	-	-	-	-	-	0.5	-
Alpha-Ketoglutaric acid	370	-	350	-	-	-	-
Fumaric acid	55	-	60	-	-	-	-
Malic acid	670	-	600	-	-	-	-
Succinic acid	60	-	60	-	-	-	-
Trehalose	-	-	2000	-	-	-	-
Yeastolate	3330	-	-	-	-	-	-
Lactalbumin hydrolysate	3303	-	-	-	-	-	-
Yeast extract	-	-	2000	-	-	-	-
Pyruvic acid	-	-	-	110	-	-	-
Ethanolamine	-	-	-	1.9	-	-	-
Tryptose broth	-	2600	-	-	-	-	-
**Other compaunds**							-
2-deoxy-D-ribose	-	-	-	-	-	0.5	-
Adenine sulfate	-	-	-	-	-	10	-
Adenosine 5'-phosphate	-	-	-	-	-	0.2	-
Adenosine 5'-triphosphate	-	-	-	-	-	1	-
2'Deoxyadenosine	-	-	-	-	10	-	-
2'Deoxycytidine	-	-	-	-	10	-	-
2'Deoxyguanosine	-	-	-	-	10	-	-
5-Methyl-deoxycytidine	-	-	-	-	0.1	-	-
Guanine hydrochloride	-	-	-	-	-	0.3	-
Thymine	-	-	-	-	-	0.3	-
Thymidine	-	-	-	0.365	10	-	-
Uracil	-	-	-	-	-	0.3	-
Co-carboxylase	-	-	-	-	1	-	-
Coenzyme A	-	-	-	-	2.5	-	-
Hypoxanthine Na	-	-	-	-	-	0.4	-
NAD	-	-	-	-	7	-	-
FAD	-	-	-	-	1	-	-
NADP	-	-	-	-	1	-	-
Uridine 5'- triphosphate	-	-	-	-	1	-	-
Cholesterol	-	-	-	-	-	0.2	-
Phenol Red	-	-	-	15	20	20	5

Sodium acetate-3H2O	-	-	-	-	83	50	-
Sodium glucuronate-H2O	-	-	-	-	4.2	-	-
Tween 80®	-	-	-	-	5	20	-
