# Supplementary material for: Cultivation reveals physiological diversity among defensive ‘Streptomyces philanthi’ symbionts of beewolf digger wasps (Hymenoptera, Crabronidae)
Source: BMC Microbiol. 2014 Jul 29;14:202. doi: 10.1186/s12866-014-0202-x (PMC4236554; doi:10.1186/s12866-014-0202-x)
Supplement: Additional file 3: Table S3. — Number of ‘S. philanthi’ CFUs isolated from different females’ antennal samples. [file s12866-014-0202-x-S3.pdf]

Table S3. Number of '*S. philanthi*' CFUs isolated from different females' antennal samples.

Geographic origin	Host species	Number of specimens			Symbionts, CFU/specimen (mean ± SE)
		Total	with symbionts isolated	with opportunistic bacteria isolated	
Europe	*Philanthus triangulum*	7	3	1	~10^4 †
South Africa	*Philanthus triangulum diadema*	11	11	0	~10^5 †
	Philanthus histrio	4	1	0	$14*10^4$
	Philanthus loefflingi	2	1	0	$2*10^4$
	Philanthus fuscipennis	3	2	1	22 ± 20
	Philanthus capensis	1	0	1	0
USA	*Philanthus albopilosus*	2	1	1	$70*10^3$
	Philanthus barbiger	2	1	1	$15*10^4$
	Philanthus bicinctus	5	5	0	$50*10^3 \pm 14*10^3$
	Philanthus bilunatus	3	3	0	$28*10^3 \pm 13*10^3$
	Philanthus crabroniformis	1	1	0	NA
	Philanthus gibbosus	3	3	0	$76*10^3 \pm 39*10^3$
	Philanthus multimaculatus	4	4	0	$43*10^3 \pm 96*10^2$
	Philanthus pacificus	1	1	0	2
	Philanthus parkeri	1	1	0	$63*10^3$
	Philanthus politus	1	1	0	$72*10^3$
	Philanthus psyche	4	4	0	$38*10^3 \pm 21*10^3$
	Philanthus pulcher	4	4	0	680 ± 447
	Philanthus ventilabris	7	6	1	$30*10^3 \pm 23*10^3$
	Philanthus zebratus	1	1	0	$1*10^4$
Brasil	*Trachypus elongatus*	1	1	0	~10^4 †
	Trachypus flavidus	3	1	0	NA †
Turkey	*Philanthinus quattuordecimpunctatus*	16	7	2	77 ± 34

NA – not assessed; † - difficult to count because micro-colonies are fuzzy and poorly structured.
