# Supplementary material for: Cultivation reveals physiological diversity among defensive ‘Streptomyces philanthi’ symbionts of beewolf digger wasps (Hymenoptera, Crabronidae)
Source: BMC Microbiol. 2014 Jul 29;14:202. doi: 10.1186/s12866-014-0202-x (PMC4236554; doi:10.1186/s12866-014-0202-x)
Supplement: Additional file 4: Table S4. — Accession numbers of actinobacterial sequences included in the phylogenetic analyses shown in Figure 3. [file s12866-014-0202-x-S4.pdf]

Table S4: Accession numbers of actinobacterial sequences included in the phylogenetic analyses shown in Fig. 3.

Genus	Species	Strain	16S	gyrB	gyrA
Frankia	*alni*	ACN14a	NC_008278	NC_008278	NC_008278
Streptomyces	*abikoensis* (=*luteoverticillatus*)	DSM40831	KC954556	KC954562	KC954559
Streptomyces	*albus*	J1074	AJ621602(1)	NZ_DS999645	NZ_DS999645
Streptomyces	*auratus*	AGR0001	-	NZ_JH725387	NZ_JH725387
Streptomyces	*avermitilis*	MA4680	NC_003155	NC_003155	NC_003155
Streptomyces	*bingchenggensis*	BCW1	NC_016582	NC_016582	NC_016582
Streptomyces	*cattleya*	NRRL8057	NC_016111	NC_016111	NC_016111
Streptomyces	*clavuligerus*	ATCC 27064	NZ_CM001015	NZ_CM001015	NZ_CM001015
Streptomyces	*coelicolor*	A3(2)	NC_003888	NC_003888	NC_003888
Streptomyces	*flavogriseus*	ATCC 33331	NC_016114	NC_016114	NC_016114
Streptomyces	*ghanaensis*	ATCC 14672	AJ781384(1)	NZ_DS999641	NZ_DS999641
Streptomyces	*griseoflavus*	Tu4000	AJ781322(1)	NZ_GG657758	NZ_GG657758
Streptomyces	*griseus subsp. griseus*	NBRC 13350	NC_010572	NC_010572	NC_010572
Streptomyces	*griseus*	XylebKG1	NZ_GL877172	NZ_GL877172	NZ_GL877172
Streptomyces	*hygroscopicus subsp. jinggangensis*	5008	NC_017765	NC_017765	NC_017765
Streptomyces	*hygroscopicus*	ATCC 53653	EF408736	NZ_ACEX01000401	ACEX01000401
Streptomyces	*lividans*	TK24	AY039029	NZ_GG657756	NZ_GG657756
Streptomyces	*mutabilis*	DSM40169	KC954557	KC954563	KC954560
Streptomyces	*pristinaespiralis*	ATCC 25486	-	NZ_CM000950	NZ_CM000950
Streptomyces	*ramulosus*	DSM40100	KC954558	KC954564	KC954561
Streptomyces	*roseosporus*	NRRL11379	NZ_ABYX01000136	NZ_ABYX01000145	ABYX01000145
Streptomyces	*scabiei*	87.22	NC_013929	NC_013929	NC_013929
Streptomyces	*sp.*	SPB78	-	NZ_GG657742	NZ_GG657742
Streptomyces	*sp.*	SPB74	-	NZ_GG770539	NZ_GG770539
Streptomyces	*sp.*	SirexAAE	NC_015953	NC_015953	NC_015953
Streptomyces	*sp.*	Tu6071	NZ_CM001165	NZ_CM001165	NZ_CM001165
Streptomyces	*sp.*	C	-	NZ_ACEW01000329	ACEW01000329
Streptomyces	*sp.*	Mg1	-	NZ_ABJF01000426	ABJF01000426
Streptomyces	*sviceus*	ATCC 29083	AB184559(1)	NZ_CM000951	NZ_CM000951
Streptomyces	*venezuelae*	ATCC 10712	NC_018750	NC_018750	NC_018750
Streptomyces	*violaceusniger*	Tu4113	NC_015957	NC_015957	NC_015957
Streptomyces	*viridochromogenes*	DSM40736	AB045858(1)	NZ_ACEZ01000135	ACEZ01000135
Streptosporangium	*roseum*	DSM 43021	NC_013595	NC_013595	NC_013595
