# Supplementary material for: Cultivation reveals physiological diversity among defensive ‘Streptomyces philanthi’ symbionts of beewolf digger wasps (Hymenoptera, Crabronidae)
Source: BMC Microbiol. 2014 Jul 29;14:202. doi: 10.1186/s12866-014-0202-x (PMC4236554; doi:10.1186/s12866-014-0202-x)
Supplement: Additional file 5: Figure S1. — AFLP-based phylogenetic analysis of cultured ‘S. philanthi’ biovars. [file s12866-014-0202-x-S5.pdf]

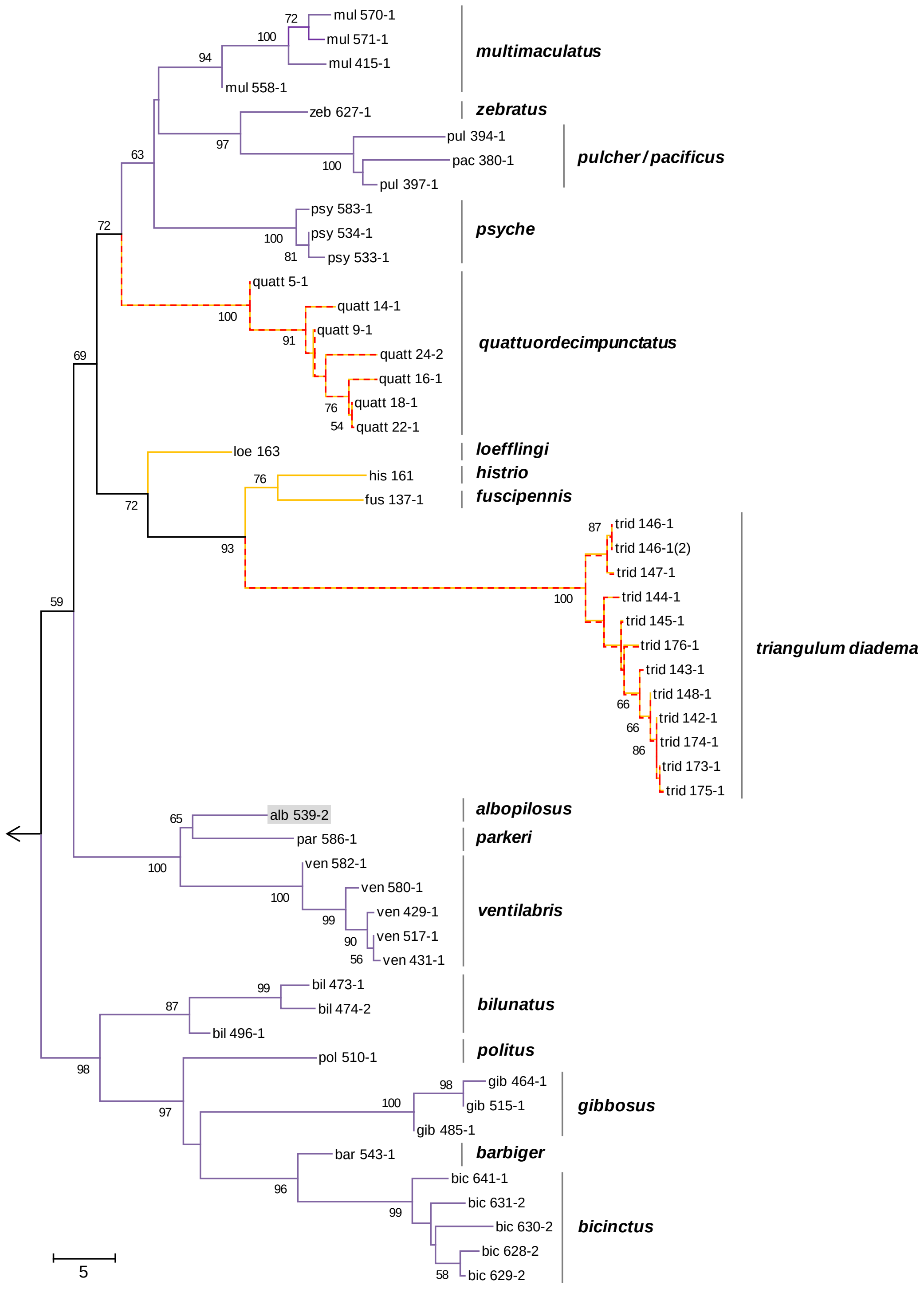

Figure S1 AFLP-based phylogenetic analysis of cultured *'S. philanthi'* biovars. Phylogenetic analysis was conducted using 202 AFLP markers (TaqI-G/-C and ApaI-CC/-CA/-GA/-GT). The evolutionary history was inferred using the Neighbor-Joining method [1]. The optimal tree with the sum of branch length = 355.93 is shown. Bootstrap values (100 replicates) above 50% are shown next to the branches. Antennal isolates are indicated by their strain designation as explained in the Methods section (first three letters indicate host species), and the respective host species is additionally given behind each clade. Geographic distribution of beewolf taxa and the origin of isolated symbionts are indicated by branches of different colours on phylogenetic tree: Africa (yellow), Europe (red), mixed African/ Eurasian distribution (red dash line), North and South America (purple and blue, respectively). Because of the discrepant phylogenetic placement of *Philanthus albopilosus* symbiont sequences from clones and isolates, respectively (see Fig. S2), biovar *albopilosus* is highlighted by a grey box.
