# Supplementary material for: Cultivation reveals physiological diversity among defensive ‘Streptomyces philanthi’ symbionts of beewolf digger wasps (Hymenoptera, Crabronidae)
Source: BMC Microbiol. 2014 Jul 29;14:202. doi: 10.1186/s12866-014-0202-x (PMC4236554; doi:10.1186/s12866-014-0202-x)
Supplement: Additional file 6: Figure S2. — Polymorphism of ‘S. philanthi’ biovars ‘elongatus’ and ‘loefflingi’. [file s12866-014-0202-x-S6.pdf]

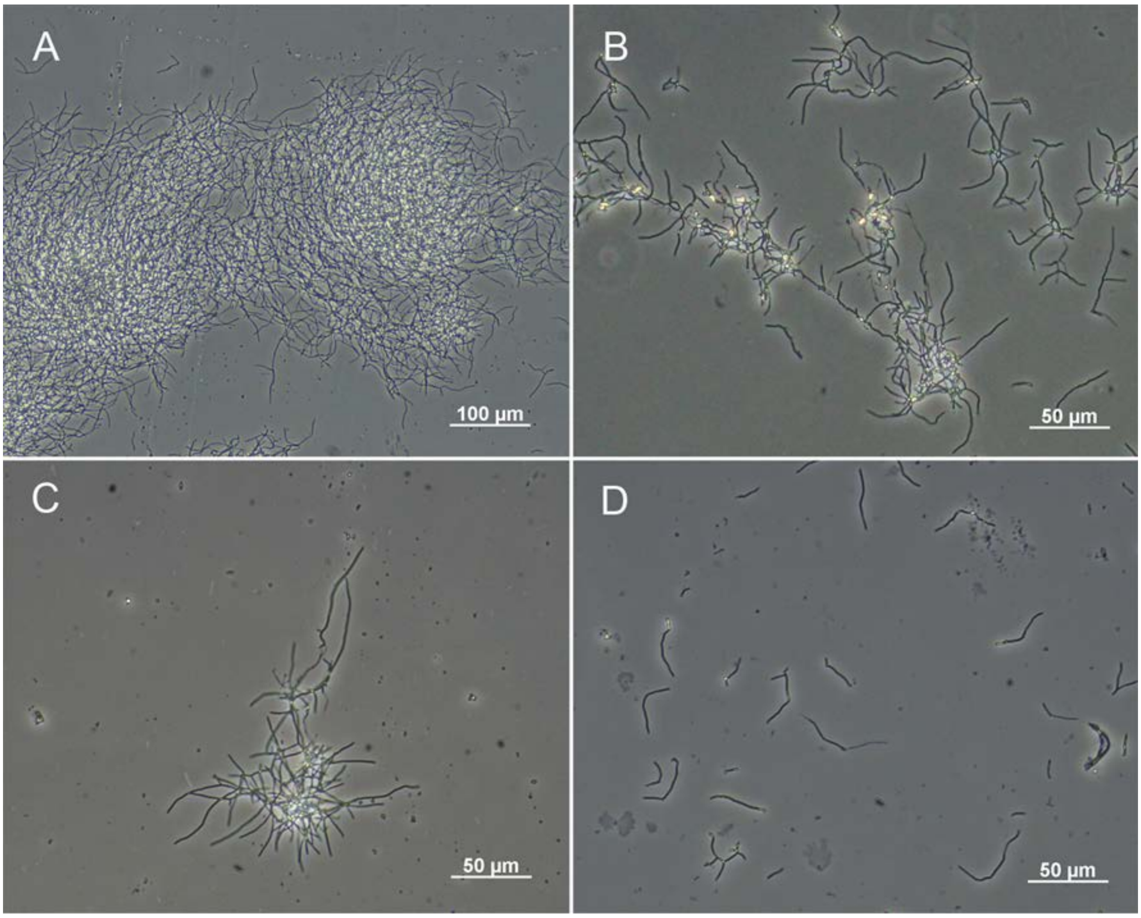

Figure S2. Polymorphism of '*S. philanthi*' biovars '*elongatus*' and '*loefflingi*'. Strains elo217 (A, B) and loe163 (C, D) (biovars '*elongatus*' and '*loefflingi*', respectively) were grown in Grace's liquid medium with 10% FBS. Bacteria of biovar '*elongatus*' formed mycelium and typical micro-colonies in early stage of logarithmic growth (A) but tend to fall apart in late stage of logarithmic growth or in stationary phase (B). Bacteria of biovar '*loefflingi*' could form micro-colonies (C) and also grow as short almost unbranched cells typical for the "antennal" phenotype (D).
