# Supplementary material for: Cultivation reveals physiological diversity among defensive ‘Streptomyces philanthi’ symbionts of beewolf digger wasps (Hymenoptera, Crabronidae)
Source: BMC Microbiol. 2014 Jul 29;14:202. doi: 10.1186/s12866-014-0202-x (PMC4236554; doi:10.1186/s12866-014-0202-x)
Supplement: Additional file 7: Figure S3. — Free-living bacteria growing on the solid modified Grace’s medium with ammonium as the only nitrogen source. [file s12866-014-0202-x-S7.pdf]

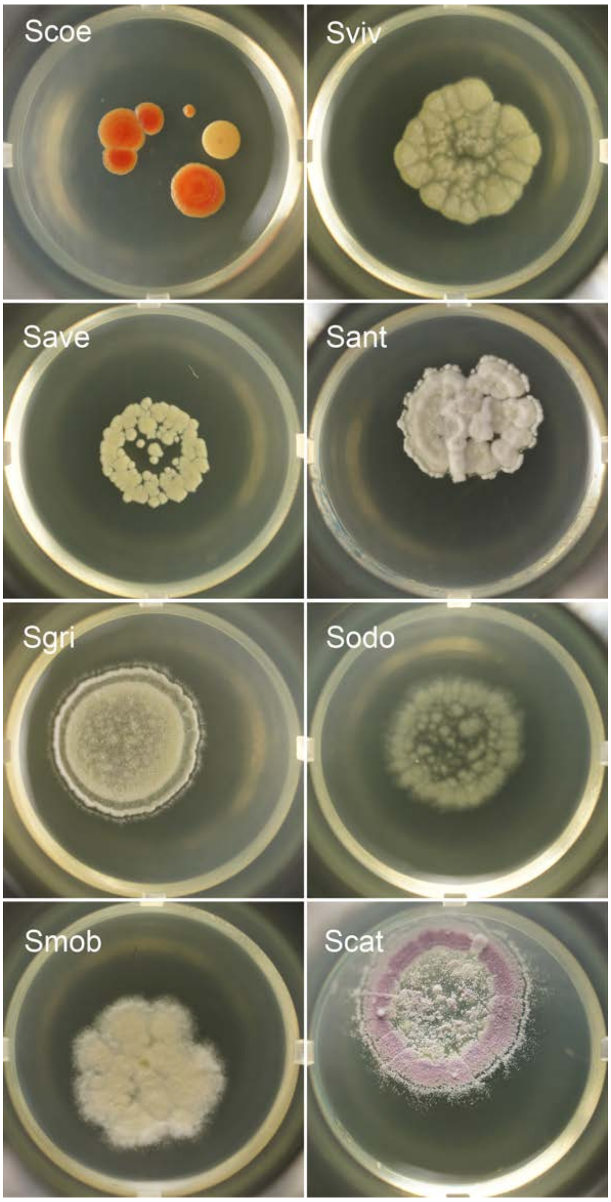

Figure S3 Free-living bacteria growing on the solid modified Grace's medium with ammonium as the only nitrogen source: *S. coelicolor* (Scoe), *S. avermitilis* (Save), *S. griseus* (Sgri), *S. mobaraensis* (Smob), *S. viridochromogenus* (Sviv), *S. antibioticus* (Sant), *S. odorifer* (Sodo), *S. cattleya* (Scat).
