# Supplementary material for: Cultivation reveals physiological diversity among defensive ‘Streptomyces philanthi’ symbionts of beewolf digger wasps (Hymenoptera, Crabronidae)
Source: BMC Microbiol. 2014 Jul 29;14:202. doi: 10.1186/s12866-014-0202-x (PMC4236554; doi:10.1186/s12866-014-0202-x)
Supplement: Additional file 8: Table S5. — Primers and adapters used for generation of AFLP markers. [file s12866-014-0202-x-S8.pdf]

**Table S5.** Primers and adapters used for generation of AFLP markers.

| **Primers/adapters** | **Sequence (5´--> 3´)** | **5´-Mod.** |
|---|---|---|
| *Adapters* | | |
| ApaI_fwd | TCGTAGACTGCGTACAGGCC | |
| ApaI_rev | TGTACGCAGTCTAC | |
| TaqI_fwd | GACGATGAGTCCTGAC | |
| TaqI_rev | CGGTCAGGACTCAT | |
| *Preamplification primers* | | |
| ApaI | GACTGCGTACAGGCCC | |
| TaqI | GATGAGTCCTGACCGA | |
| *Selective amplification primers* | | |
| TaqI-G_IR700 | GATGAGTCCTGACCGAG | IRD700 |
| TaqI-C_IR800 | GATGAGTCCTGACCGAC | IRD800 |
| ApaI-CA | GACTGCGTACAGGCCCCA | |
| ApaI-CC | GACTGCGTACAGGCCCCC | |
| ApaI-CG | GACTGCGTACAGGCCCCG | |
| ApaI-CT | GACTGCGTACAGGCCCCT | |
| ApaI-GA | GACTGCGTACAGGCCCGA | |
| ApaI-GC | GACTGCGTACAGGCCCGC | |
| ApaI-GG | GACTGCGTACAGGCCCGG | |
| ApaI-GT | GACTGCGTACAGGCCCGT | |
| ApaI-AA | GACTGCGTACAGGCCCAA | |
| ApaI-AC | GACTGCGTACAGGCCCAC | |
